# Comparison of Diagnostic Performance of Spread Through Airspaces of Lung Adenocarcinoma Based on Morphological Analysis and Perinodular and Intranodular Radiomic Features on Chest CT Images

**DOI:** 10.3389/fonc.2021.654413

**Published:** 2021-06-25

**Authors:** Lin Qi, Xiaohu Li, Linyang He, Guohua Cheng, Yongjun Cai, Ke Xue, Ming Li

**Affiliations:** ^1^ Department of Radiology, Huadong Hospital Affiliated to Fudan University, Shanghai, China; ^2^ Department of Radiology, First Affiliated Hospital of Anhui Medical University, Hefei, China; ^3^ Jianpei Technology Co., Ltd., Hangzhou, China; ^4^ Department of Pathology, Huadong Hospital Affiliated to Fudan University, Shanghai, China; ^5^ Department of Plastic and Reconstructive Surgery, Shanghai 9th Peoples Hospital Affiliated to Shanghai Jiaotong University School of Medicine, Shanghai, China

**Keywords:** lung, adenocarcinoma, radiomics, spread through airspaces, machine learning

## Abstract

**Object:**

STAS is associated with poor differentiation, KRAS mutation and poor recurrence-free survival. The aims of this study are to evaluate the ability of intra- and perinodular radiomic features to distinguish STAS at non-contrast CT.

**Patients and Methods:**

This retrospective study included 216 patients with pathologically confirmed lung adenocarcinoma (STAS+, n = 56; STAS−, n = 160). Texture-based features were extracted from intra- and perinodular regions of 2, 4, 6, 8, 10, and 20 mm distances from the tumor edge using an erosion and expansion algorithm. Traditional radiologic features were also analyzed including size, consolidation tumor ratio (CTR), density, shape, vascular change, cystic airspaces, tumor–lung interface, lobulation, spiculation, and satellite sign. Nine radiomic models were established by using the eight separate models and a total of the eight VOIs (eight-VOI model). Then the prediction efficiencies of the nine radiomic models were compared to predict STAS of lung adenocarcinomas.

**Results:**

Among the traditional radiologic features, CTR, unclear tumor–lung interface, and satellite sign were found to be associated with STAS significantly, and the AUCs were 0.796, 0.677, and 0.606, respectively. Radiomic model of combined tumor bodies and all the distances of perinodular areas (eight-VOI model) had better predictive efficiency for predicting STAS+ lung adenocarcinoma. The AUCs of the eight-VOI model in the training and verification sets were 0.907 (95%CI, 0.862–0.947) in the training set, and 0.897 (95%CI, 0.784–0.985) in the testing set, and 0.909 (95%CI, 0.863–0.949) in the external validation set, and the diagnostic accuracy in the external validation set was 0.849.

**Conclusion:**

Radiomic features from intra- and perinodular regions of nodules can best distinguish STAS of lung adenocarcinoma.

## Introduction

In 2015, the World Health Organization (WHO) formally proposed a new mode of invasion of lung cancer: spread through air spaces (STAS), defined as “micropapillary clusters, solid nests or single cells spreading within air paces beyond the edge of the main tumor” (1). STAS can be considered the same as other more common invasive growth patterns, such as vessels or pleural infiltration (2). It is associated with poor differentiation, K-RAS mutation, and poor recurrence-free survival (RFS). Free-floating tumor cells or tumor clusters can survive in the alveolar cavity for a long time. Studies have confirmed (3–5) that STAS is an important prognostic indicator for early lung adenocarcinoma, and the way to reduce the recurrence rate of early lung cancer after STAS is to change localized resection to a lobectomy and postoperative adjuvant chemotherapy. Therefore, if STAS can be detected before surgery, it can provide important information for the choice of resection method and whether adjuvant chemotherapy is given to patients with early lung adenocarcinoma after surgery. Current studies (6, 7) have shown that frozen pathological sections before surgery cannot diagnose STAS, and there is also a lack of reliable CT signs for preoperative diagnosis.

At present, radiomics studies for STAS are mainly based on feature extraction and modeling of the main body of the lesion, while STAS is mainly spread around the edge of the lesion. We speculated that the perinodular area may possess some valuable information to improve the efficiency of intranodular radiomic analysis. Therefore, this study attempted to evaluate whether the radiomics features of the combination of intra- and perinodular areas together were more predictive of STAS than the intranodular radiomic model or the morphological analysis of CT signs alone.

## Patients And Methods

### Patients

A total of 512 patients with primary lung adenocarcinoma confirmed by surgical resection and pathology were collected continuously and retrospectively at our institution from January 2017 to May 2020. The interval between CT scan and surgery is within 2 months. Patients with the following criteria were excluded: atypical hyperplasia and adenocarcinoma *in situ* (n = 172), lack of preoperative non-contrast CT images or obvious CT artifacts (*n* = 37), previous lung surgery or preoperative adjuvant chemotherapy (*n* = 32), pathological diameter >5 cm (*n* = 38), and mucinous adenocarcinoma and mucinous carcinoma (*n* = 17). There were 216 patients in the final cohort (males, 125), which included 56 STAS+ and 160 STAS− adenocarcinomas. This retrospective study was approved by the institutional review committee and the requirement for informed consent was waived.

To evaluate the performance of the best radiomic model, we used 46 consecutive external data from another hospital as the testing set in accordance with the aforementioned inclusion criteria. The inclusion and exclusion criteria of the external set were consistent with that of the cohort collected in our center. The external data included 15 STAS+ adenocarcinoma (eight men; mean age, 52 ± 8.7 years) and 31 STAS− adenocarcinoma(21 men; mean age, 54 ± 7.6 years).

### CT Acquisition and Morphologic Evaluation

CT images were acquired from General Electric (LightSpeed VCT; Waukesha, Wis) or Siemens (Definition Flash, Erlangen, Germany). CT parameters were as follows: tube voltage, 120 kVp; tube current, 150–200 mAs; pitch, 0.984:1 or 1.0; reconstructed thickness and interval, 1.25 mm or 1 mm. All images were reviewed and measured with a standard lung window (window width, 1500 HU; window level, −500 HU) and a mediastinal window (window width, 350 HU; window level, 50 HU).

The size of the entire lesion was defined as the average of long and short axial diameters. The consolidation tumor ratio (CTR) was defined as the proportion of the maximum consolidation diameter divided by the maximum tumor diameter. The CT morphologic features were evaluated as follows: nodule density (solid, part solid, or pure ground glass), location (upper or not), and shape (round to oval, irregular), vascular change (normal, convergent or dilated), cystic airspaces, tumor-lung interface (clear or unclear), lobulation, spiculation, and satellites around the lesion. Morphological analysis was performed by two radiologists with 10 and 20 years of experience in chest radiologic diagnosis (LQ and ML), and they were blinded to the pathological results. Any disagreements between them were resolved by reaching a consensus.

### Pathological Analysis

Surgically resected specimens were fixed with formalin and stained with hematoxylin–eosin. According to the 2015 WHO classification (1), STAS was defined as micropapillary clusters, solid nests, or single cells spread within the air spaces beyond the edge of the main tumor. Tumor cells that spread through the mucus were distinguished from STAS and excluded from our study.

### Data Preparation for Radiomics

Digital Imaging and Communications in Medicine (DICOM) images were downloaded from the picture archiving and communication system (PACS). An open-source medical image processing and navigation software (3D Slicer, version 4.8.0; National Institutes of Health; http://www.slicer.org) were used for pixel-level labeling of pulmonary nodules by one radiologist with 10 years of experience in lung cancer diagnosis; then, the labeling results were confirmed by another radiologist with 20 years of CT diagnostic experience. The structures of blood vessels, bronchi, and pleura outside the nodules were excluded as much as possible, and the three-dimensional VOIs of the tumors were constructed.

A total of 216 cases were manually labeled and reviewed, and the volume VOI of the lesion and all the CT data were exported in NII format for subsequent analysis. Each case was accompanied by a STAS label without any additional information, such as sex, age, location, morphological features. We randomly divided 80% of each of the two groups as training data sets (n = 172, 46 in STAS+ group, 126 in STAS− group) and the rest as verification data sets (n = 44, 10 in STAS+ group, 34 in STAS− group). The training set was used to train the designed group model, and the verification set was used to evaluate the accuracy of the model.

### Intra- and Perinodular Segmentation

**Figure 1:**
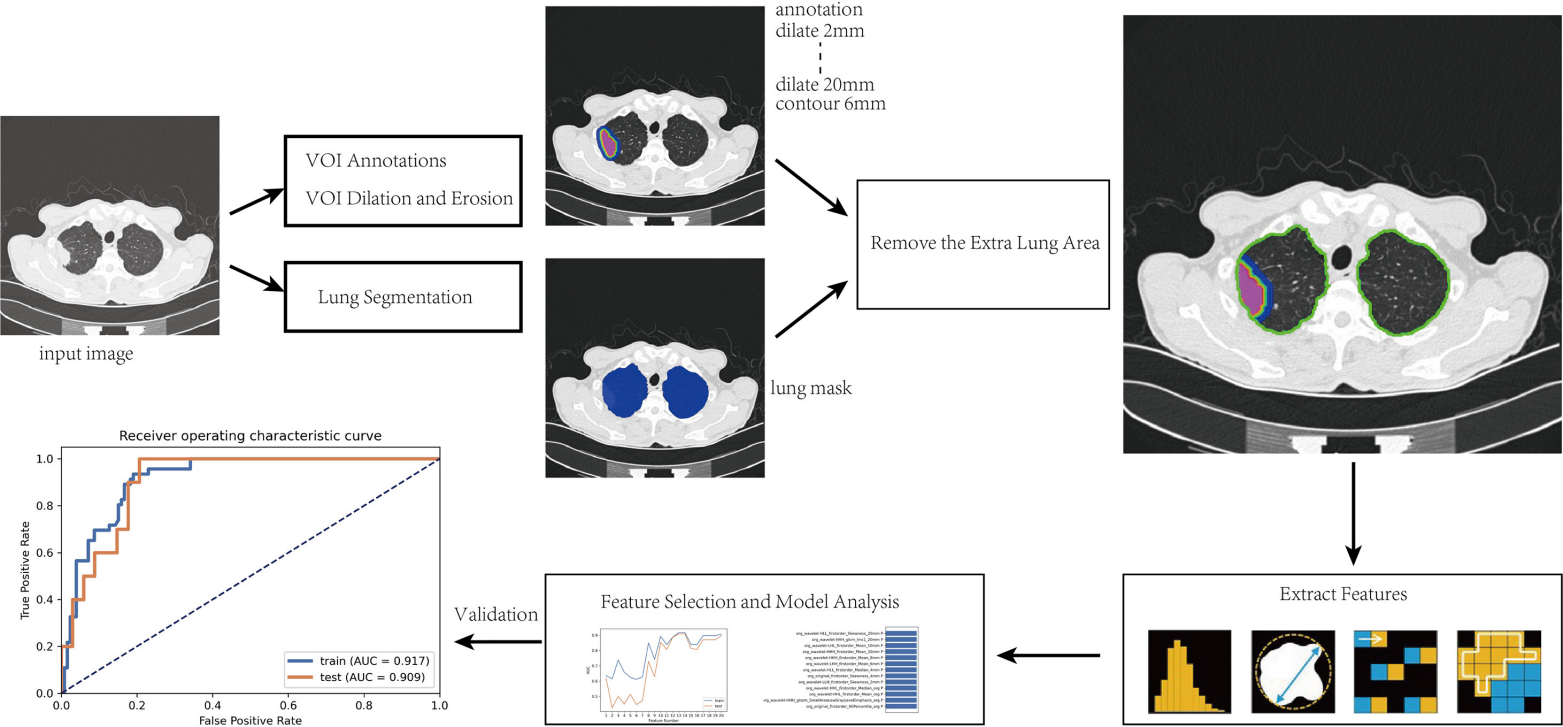
Flowchart of radiomics procedure in this study.

**Figure 2:**
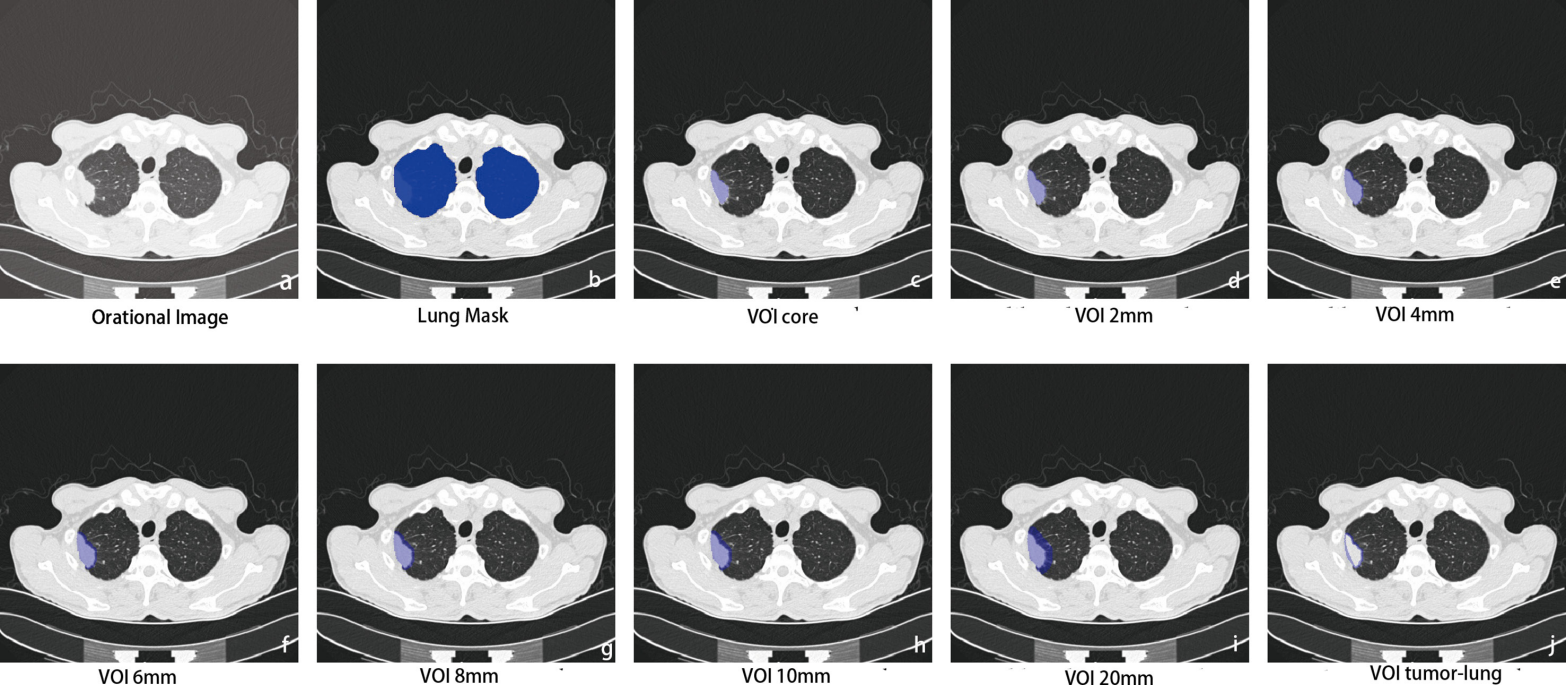
Intra- and perinodular segmentation. Erosion and expansion algorithm programs were used to capture the perinodular area, and then the intranodular mask was subtracted from the expanded mask to obtain perinodular areas from the tumor surface. **(A)** Plan CT image shows subpleural solid nodules in the upper lobe of the right lung; **(B)** lung tissue segmentation mask: perinodular area was dilated within this area to remove the extrapulmonary structures; **(C)** manual labeling to obtain the original 3D region of interest as the core area (VOI_core_), which was used as a seed to expand outward within the lung tissue mask. **(D–I)** The different distances of 3D regions of interest from the tumor surface were obtained at 2, 4, 6, 8, 10, and 20 mm (marked as VOI_2–20 mm_); **(J)** the marginal regions of each 3 mm inside and outside the contour of VOI_core_ were annotated (VOI_tumor–lung_).

After the intranodular mask was annotated, a program was written to expand to the surrounding area within the lung tissue to capture the perinodular region up to a maximum distance of 20 mm. The modeling steps and radiomic analysis process are as follows and shown in **Figure 1**:

(1) The manually annotated three-dimensional (3D) volume of interest (VOI) was taken as the core area (marked as VOI _core_), and lung segmentation was used to exclude areas outside the lung tissue to prepare for obtaining the perinodular area;
(2) Effective features were screened by feature engineering and then modeled by Adaboost. Python 3.6.1 (https://www.python.org) was used to write the erosion and expansion algorithm program to capture the perinodular area, and then the intranodular mask was subtracted from the expanded mask to obtain perinodular areas from the tumor surface. The different distances of 3D regions of interest from the tumor surface were obtained as 2, 4, 6, 8, 10, 20 mm (marked as VOI_2 mm_, VOI_4 mm_, VOI_6m m_, VOI_8 mm_, VOI_10 mm_, and VOI_2 0mm_, respectively);
(3) By using the method of step (2), the marginal regions of each 3 mm inside and outside the contour of the VOI_core_ were annotated representing the 3 mm area of the tumor shell plus the 3 mm peripheral area including the tumor–lung interface (marked as VOI_tumor–lung_).
Finally, we obtained a total of eight different VOI areas, which were combined with the mask of lung segmentation, and the extrapulmonary regions were excluded (**Figure 2**).

### Feature Extraction and Analysis

The PyRadiomics software package *(* http://www.radiomics.io/pyradiomics.html *)* was used to extract imaging features from each VOI, including first-order features, morphological features, gray co-occurrence matrix (GLCM), gray run matrix (GLRLM), gray area size matrix (GLSZM), gray co-occurrence matrix (GLDM) texture features, and wavelet frequency domain texture features. Then, 851 features were extracted from each VOI, and a total of 6,808 imaging features (https://pyradiomics.readthedocs.io/en/latest/features.html#) were calculated for each CT image input, and the Z-score was standardized. To eliminate the influence of sample class imbalance on training, the synthetic minority class oversampling technique (Synthetic Minority Oversampling Technique, SMOTE) was used to maintain the balance between positive samples and negative samples. Because the dimension of the feature space is very high, we compared the similarity of each set of feature pairs. The Pearson correlation coefficient (Pearson Correlation Coefficient, PCC) was used to evaluate the correlation degrees of the extracted imaging features, which could reflect the linear correlation degree of the two variables. PCC is defined as the entropy of covariance and standard deviation between two characteristic variables. The calculation formula is as follows:

$$\rho_{xy}=\frac{Cov\left(X,Y\right)}{\sigma_{x}\sigma_{y}}=\frac{E\left[\left(X-\mu_{x}\right)\left(Y-\mu_{y}\right)\right]}{\sigma_{x}\sigma_{y}}\mu_{i}\sigma_{i}$$

Among them, *X* and *Y* are the two characteristic variables; *µ*_i_ and *σ_i_* were respectively.

If the PCC of a set of feature pairs was greater than 0.99, one of them was removed. After the dimension reduction of the Pearson correlation coefficient, the feature dimension of the feature space was reduced, and each feature was independent of each other. Before establishing the model, we used the recursive feature elimination method (recursive feature elimination, RFE) to evaluate the prediction efficiency of the selected features and further screened out the features with predictive value.

The goal of RFE is to select features based on classifiers by recursively considering smaller feature sets. AdaBoost was used as the classifier in this study. The basic principle of the Adaboost algorithm is to combine multiple weak classifiers into a strong classifier. AdaBoost is sensitive to noise and outliers, which can also avoid overfitting. Here, we used the decision tree as the basic classifier. To determine the superparameters of the model (such as the number of features), five-fold cross-validation was used for the training data set. Superparameters were set based on the performance of the model on the validated dataset.

Nine radiomic models were established by using the eight separate models (VOI_2 mm_, VOI_4 mm_, VOI_6 mm_, VOI_8 mm_, VOI_10 mm_, VOI_20 mm_, and VOI_tumor–lung_) and a total of the eight VOIs (eight-VOI model). Then the prediction efficiencies of the nine radiomic models were compared. First, the extracted features were grouped according to different categories, including first-order features and morphological features, texture features (GLCM, GLRLM, GLSZM, and GLDM), and wavelet frequency features, and each set of features was used for modeling separately. Then, all the radiomic features were used for modeling. The grid search was used to select model hyperparameters, and the best hyperparameters were reversely selected based on the AUC in the validation set. For each model, 50% discount cross-validation was used for training. The probabilities of model prediction in the training and verification sets in each iteration were recorded, and the mean value of the probability recorded in each iteration when each data point was used as the training set or verification set was calculated as the result of the training set or verification set of the model.

The effectiveness of CT-based imaging radiomics in predicting STAS was evaluated by using the area under the receiver operating characteristic curve (ROC) metric. The area under the curve (AUC), sensitivity, specificity, accuracy, positive predictive value (PPV), and negative predictive value (NPV) were calculated under the critical value of maximizing the Youden index. We also estimated the 95% confidence interval of 1,000 samples by bootstrapping. Significance was defined as probability values less than 0.05 (*p-value* < 0.05).

### Statistical Analysis

Statistical analysis was performed by SPSS software (version 22.0, IBM SPSS Statistics, Armonk, NY, USA). Quantitative data were expressed as mean ± standard deviation. The clinical, pathological, and CT traditional morphological features of STAS+ and STAS− groups were compared using Shapiro–Wilk test or Mann–Whitney U test. Chi-square test was used to compare the differences of classified variables.

ROC curves were drawn to compare the diagnostic efficiencies of different CT signs. MedCalc software (Version19.0.2) was used to calculate the diagnostic efficacy of classification variables for STAS, and the ROC curve of the histological model was tested by DeLong to compare whether the efficiency difference among the models was statistically significant.

## Results

### Clinical Data and Traditional Imaging Features

The basic clinical data and CT morphological characteristics of the two groups are shown in **Table 1**. Among the 216 patients with lung adenocarcinoma (male, 58.9%; mean age, 56 ± 11 years), 56 patients were confirmed to be STAS+ pathologically (male, 62.5%; mean age 55 ± 9 years), and 160 patients were STAS− (male, 56.3%, mean age, 56 ± 10 years). Among all patients enrolled, 78 patients (36.1%) underwent sublobectomy, and 138 (63.9%) underwent lobectomy or pneumonectomy. There was no significant difference in age, sex, or the proportion of heavy smokers between the two groups. The proportion of patients who underwent sublobectomy in the STAS− group was slightly higher than that in the STAS+ group (*p* = 0.044).

**Table 1:** The basic clinical data and CT morphological characteristics of the two groups.

	Total (n = 216)	STAS+ (n = 56)	STAS− (n = 160)	z value	*p*-value
Age (years)	56 ± 11	55 ± 9	56 ± 10	1.772	0.100
Male, n [%]	125 [58.9]	35 [62.5]	90 [56.3]	0.815	0.415
Heavy smoke, n [%]	67 (31)	16 [28.6]	51 [31.9]	0.460	0.646
Surgery				2.011	0.044^*^
Sublobar resection, n [%]	78 [36.1]	14 (25)	64 (40)		
Lobectomy or pneumonectomy, n [%]	138 [63.9]	42 (75)	96 (60)		
Pathology					<0.0001^****^
Predominant histologic subtypes					
Lepidic, n [%]	35 [16.2]	0	35 [21.9]		
Acinar, n [%]	80 (37)	5 [8.9]	75 [46.9]		
Papillary or micropapillary, n [%]	58 [26.9]	31 [55.4]	27 [16.9]		
Solid, n [%]	33 [15.3]	16 [28.6]	17 [10.6]		
Cribriform, n [%]	10 [4.6]	4 [7.1]	6 [3.8]		
Vascular invasion (+), n [%]	57 [26.4]	16 [28.6]	41 [25.6]	2.207	0.332
Pleural invasion (+), n [%]	50 [23.1]	24 [42.8]	26 [16.3]	19.49	<0.0001^****^
EGFR mutation (+), n [%]	97 [41.1]	15 [26.8]	83 [51.9]	13.16	0.001^**^
Morphological features					
Size (mm)	19.7 ± 6.2	20.2 ± 6.1	17.6 ± 6.7	173.5	0.186
CTR	0.7 ± 0.3	0.8 ± 0.3	0.5 ± 0.3	90.5	<0.0001^****^
Nodule density				11.35	0.010^**^
pGGN, n [%]	15 [6.9]	0	15 [9.4]		
mGGN, n [%]	106 [4.6]	24 [7.1]	82 [3.8]		
SN, n [%]	95 [26.4]	32 [28.6]	63 [25.6]		
Location				3.862	0.145
Upper lobes, n [%]	109 [50.5]	24 [42.9]	85 [53.1]		
Non-upper lobes, n [%]	107 [49.5]	32 [57.1]	75 [46.9]		
Shape				104.4	<0.0001^****^
Round or oval, n [%]	81 [37.5]	29 [51.8]	52 [32.5]		
Irregular, n [%]	135 [62.5]	27 [48.2]	108 [67.5]		
Vascular change, n [%]	179 [82.9]	44 [78.6]	135 [84.4]	3.052	0.217
Cystic airspaces, n [%]	78 [36.1]	13 [23.2]	65 [40.6]	7.780	0.020^*^
Edge features, n [%]					
Unclear tumor–lung interface, n [%]	90 [41.7]	38 [67.9]	52 [32.5]	24.60	<0.0001^****^
Lobulation, n [%]	98 [45.4]	22 [39.3]	76 [47.5]	3.206	0.020
Spiculation, n [%]	119 [55.1]	36 [64.3]	83 [51.9]	4.744	0.093
Satellites, n [%]	20 [9.3]	14 (25)	6 [3.8]	25.61	<0.0001^****^

STAS, spread through air spaces; EGFR, epidermal growth factor receptor; CTR, consolidation tumor ratio; pGGNs, pure ground glass nodules; mGGNs, mixted ground glass nodules; SNs, solid nodules. Significant level marks: *p < 0.05, **p< 0.01, ****p< 0.0001.

There was a significant difference in the histological subtypes of lung adenocarcinoma between the STAS+ and STAS− groups (p < 0.0001): papillary, micropapillary, and solid types were predominant in STAS+ adenocarcinoma, while lepidic and acinar subtypes were more common in the STAS− group. In addition, the incidence of pleural invasion was higher, and the incidence of EGFR mutation in STAS+ lung adenocarcinoma was lower than that in STAS− adenocarcinoma. The incidence of pleural invasion and EGFR mutation was higher in STAS+ adenocarcinoma.

No difference was observed in the average diameter of the lesions between STAS+ and STAS− tumors, but the CTR of STAS+ tumors was greater than that in STAS− tumors (0.8 *vs* 0.5, *p* < 0.0001), and the proportion of solid nodules was higher in STAS+ cases (28.6 *vs* 25.6%, p = 0.01). Among the traditional morphological features, STAS+ lung adenocarcinoma showed more round or oval nodules, unclear tumor–lung interface and satellite focus (p < 0.0001), while STAS− lesions showed more cystic airspaces (p < 0.020).

### Diagnostic Efficiency of Traditional Imaging Features

CTR achieved an AUC of 0.796 (a sensitivity of 0.915 and a specificity of 0.686) for predicting STAS, and the optimal critical value was 0.88. Among the traditional morphological CT features, “unclear tumor–lung interface” and “satellite sign” achieved higher diagnostic performance to predict STAS with AUCs of 0.677 and 0.606, respectively. The AUCs of “solid nodule”, “irregular shape”, and “vacuole sign” were all less than 0.6, and the accuracy was less than 60% (**Table 2**).

**Table 2:** Diagnostic performances of traditional radiologic features of STAS+ lung adenocarcinoma.

	AUC	Sensitivity	Specificity	Accuracy	PPV	NPV
Solid nodule	0.589	0.61	0.57	0.60	0.34	0.80
Irregular shape	0.404	0.48	0.33	0.37	0.20	0.64
Cystic airspaces	0.413	0.23	0.59	0.50	0.17	0.69
Unclear tumor-lung interface	0.677	0.68	0.68	0.68	0.42	0.86
Satellites	0.606	0.25	0.96	0.78	0.70	0.79

AUC, area under the curve, PPV, positive predictive value, NPV, negative predictive value.

### Feature Selection, Model Construction, and Validation

There was no significant difference between the training set and verification set of the main clinical data (*i.e.*, age, sex, size, CTR, and pathological subtypes) in the STAS+ and STAS− groups. Therefore, it is considered that the case distribution of the training set and verification set is balanced, which is suitable for establishing and verifying the histological analysis model.

**Figure 3:**
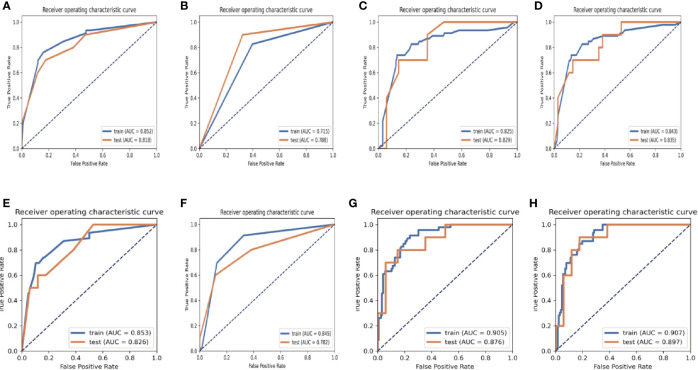
ROC graphs in the eight-VOI and VOI_core_ Models. **(A–D)** showing the ROC curves of the training and validation sets obtained by using first-order features and morphological features **(A)**, texture features **(B)**, wavelet frequency domain features **(C)** and all three kinds of features **(C)** of the VOI _core_ Model; **(E–H)** showing the ROC curves of those in eight-VOI model.

**Figure 4:**
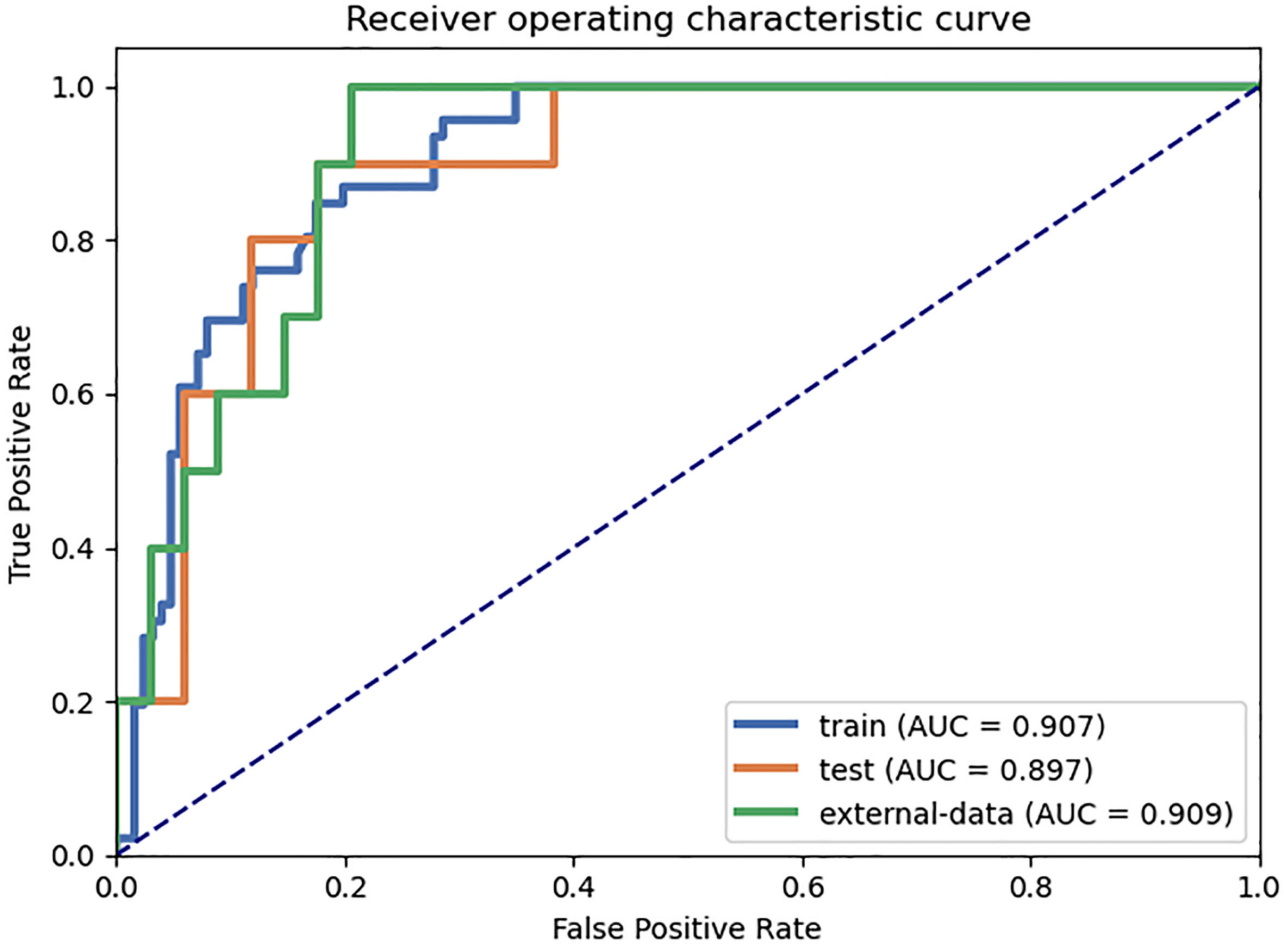
The eight-VOI radiomic model achieved the best diagnostic performance. The AUCs of the eight-VOI model were 0.907 (95%CI, 0.862–0.947) in the training set, 0.897 (95%CI, 0.784–0.985) in the testing set, and 0.909 (95%CI, 0.863–0.949) in the external validation set, and the diagnostic accuracy in the external validation set was 0.849.

The results of the diagnostic efficiencies of the nine radiomic models are shown in **Table 3**. We extracted 851 features in each model and compared the AUCs in the training and testing datasets respectively. The VOI_core_ model achieved the best diagnostic efficiency among the eight separate radiomic models and the AUCs of the training and testing sets were 0.843 (95% CI, 0.772–0.908) and 0.835 (95% CI, 0.682–0.963), respectively, and the diagnostic accuracy in the validation set was 0.818. The performances of eight separate radiomic models were significantly lower than that of eight-VOI model. The eight-VOI model achieved the best results by using all feature modeling (**Table E1**). All 6,808 imaging features were modeled jointly, and 20 radiomic features were incorporated in the model after feature selection (**Figure 3**). The AUCs of the eight-VOI model were 0.907 (95%CI, 0.862–0.947) in the training set, 0.897 (95%CI, 0.784–0.985) in the testing set, and 0.909 (95%CI, 0.863–0.949) in the external validation set, and the diagnostic accuracy in the external validation set was 0.849 (**Figure 4**).

**Table 3:** Diagnostic performances of the eight separate models and the 8-VOI model.

Radiomic Model	AUC train	AUC test
VOI_2 mm_	0.861	0.762
VOI_4 mm_	0.841	0.826
VOI_6 mm_	0.88	0.778
VOI_8 mm_	0.846	0.801
VOI_10 mm_	0.821	0.828
VOI_20 mm_	0.851	0.834
VOI_tumor–lung_	0.856	0.837
VOI_core_	0.843	0.835
Eight-VOI model	0.907	0.897

The modeling performance of a single texture feature was the worst, and the modeling effects of any other single feature type were relatively poor. Compared with the VOI_core_ model, eight-VOI model was generally improved, and the wavelet features had the greatest contribution.

**Figure 5:**
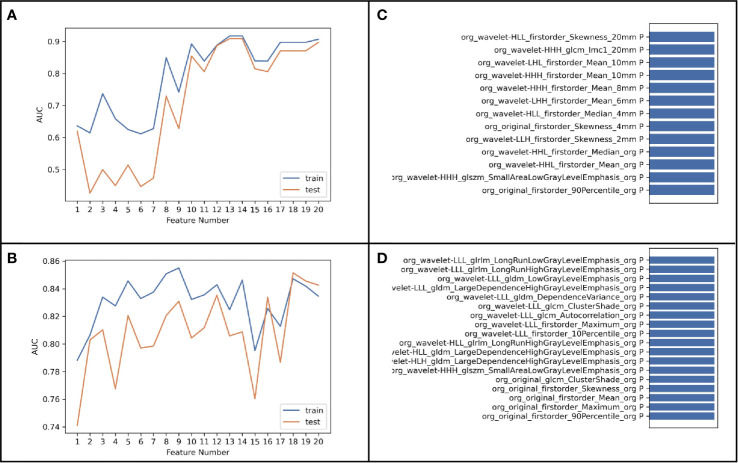
**(A, B)** was the result of modeling using 6,808-dimensional features extracted in eight-VOI Model, and **(C, D)** was the result of using 851-dimensional features extracted only in VOI_core_ model.

**Figure 5** shows the results of modeling using 6,808- and 851-dimentional features extracted in eight-VOI model and VOI_core_ model, respectively. The best performance of VOI_core_ model was obtained by using all three types of feature modeling. Compared with the VOI_core_ model alone, the best performance of eight-VOI modeling method was 7.4 and 2.8% higher in the AUC and accuracy of the verification set, respectively. Among the 20 radiomic features of the eight-VOI model (**Table E2**), there were five VOI_core_ features, two VOI_2 mm_ and VOI_4 mm_ features, three VOI_6 mm_ and VOI_8 mm_ features, and two VOI_20 mm_ features (**Figure 4**). The perinodular features accounted for 75% of the total effective features, which suggests that the radiomic features of perinodular tissues were crucial to predict STAS+ adenocarcinoma.

## Discussion

This study analyzed the predictive value of traditional imaging features, radiomic features of the tumor body and the surrounding areas in the differential diagnosis of STAS+ lung adenocarcinoma. In this study, we extracted the perinodular areas around the tumor at different distances by using the erosion and expansion algorithm for the first time and compared the diagnostic efficiencies of intra- and perinodular radiomic models in the diagnosis of STAS.

We found that radiomic model of combined tumor bodies and different distances of perinodular areas had better predictive efficiency for predicting STAS+ lung adenocarcinoma (the verification set AUC was 0.835, and the accuracy was 0.818), which was 7.4 and 2.8% higher than that of the tumor body model alone. This study explored the performance of different types of radiomic models and found that the wavelet frequency domain feature model was effective in predicting STAS, and the radiomic model combining the three types of features had the best predictive performance. We suggest that radiomics could effectively predict STAS in lung adenocarcinoma. In addition, this study found that radiomic features had better predictive effectiveness compared to traditional morphological features. It may be a promising imaging biomarker for predicting STAS before surgery and may be helpful for surgeons to choose correct operation method to reduce the postoperative recurrence rate.

**Figure 6:**
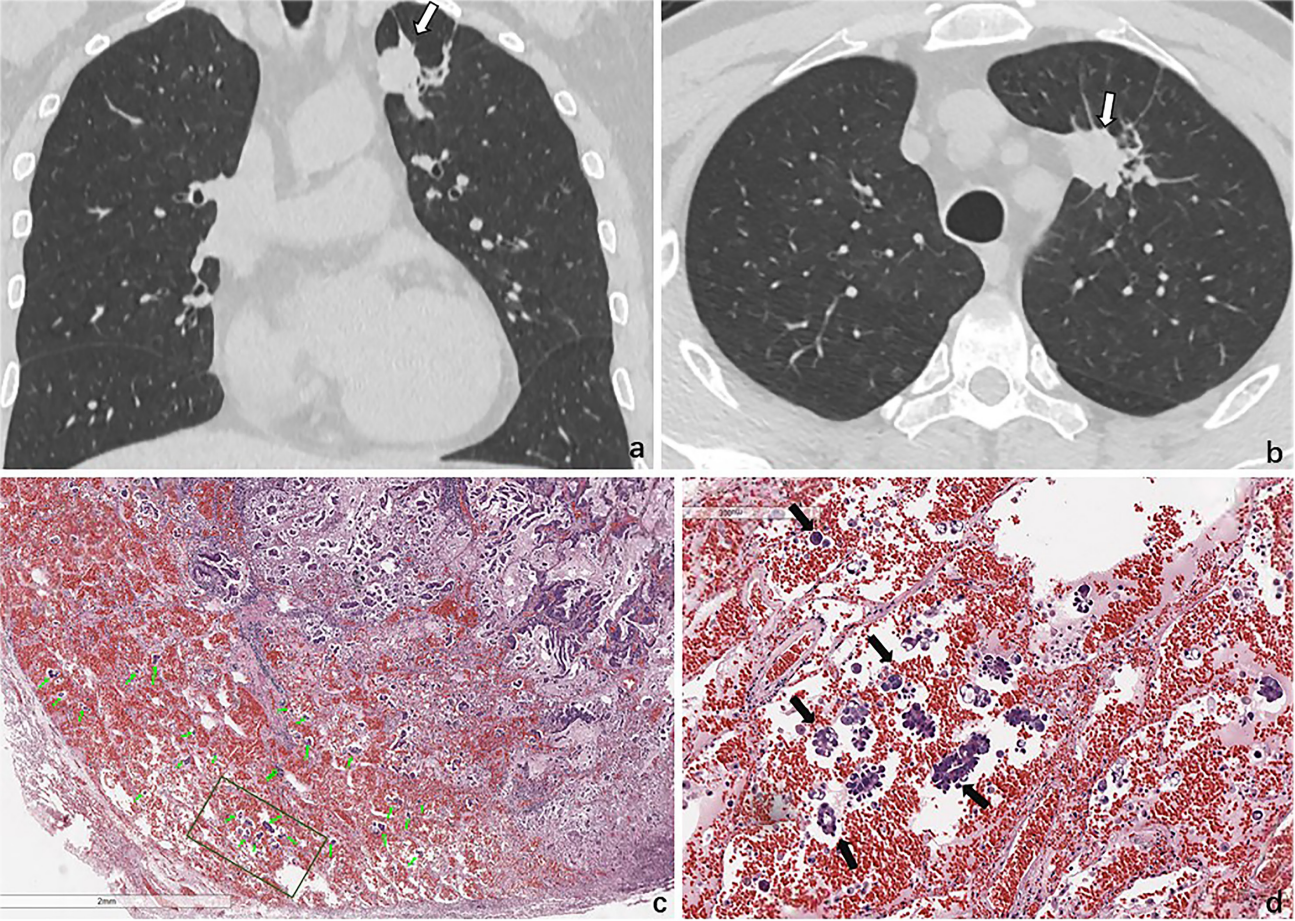
STAS in a 47-year-old man with invasive adenocarcinoma. Baseline coronal **(A)** and axial **(B)** CT images show a solid nodule in the upper lobe of the left lung. **(C)** Photomicrograph shows multiple tumor cells spread though alveolar spaces (green arrows). **(D)** They are nestlike and distrusted in the alveolar cavity around the tumor (black arrows).

The majority of studies (6, 8, 9) used in diagnosing STAS+ adenocarcinoma have focused solely on the analysis of traditional radiologic features and tumor body texture analysis. Our findings were in consensus with Kim et al. (10), who showed that CTR was independently associated with STAS in lung adenocarcinoma (AUC, 0.77; cutoff value, 90%), and none of the other CT features was associated with STAS. de Margerie-Mellon et al. (11) found that STAS+ adenocarcinoma had a higher proportion of solid components. In our cohort, the AUC and cutoff values were 0.796 and 0.88, respectively, which suggests that even small solid lesions are more likely to spread through air spaces than larger subsolid tumors with higher percentage of ground glass components. This is in line with the recommendations of the National Comprehensive Cancer Network guidelines for the treatment of non-small-cell lung cancer, which suggests that subsegmental resection is feasible for small nodules with ground glass composition greater than 50% on CT (12). We found that other imaging signs could also predict STAS in lung adenocarcinoma, including unclear tumor–lung interfaces, satellite signs and vacuole signs, but their predictive power was lower than that of CTR. Studies (13, 14) (15) on CT morphologic analysis of lung cancer showed that an irregular or cloudy surface on CT scan was highly associated with the presence of at least a feature of aggressive local spread, and there was an obvious relationship between the smooth and clear appearance of lung nodules on CT scan and smooth microscopic appearance, which was equivalent to the absence of aggressive local spread (16). Although we found that STAS+ lung adenocarcinoma had a higher probability of satellites near the main tumor, this macroscopic sign has nothing to do with STAS, which is a microscopic sign and cannot be recognized by CT scanners at present (**Figure 6**). Therefore, the predictive CT signs reported in the current research are all indirect signs, and incalculable misdiagnosis and overdiagnosis are inevitable using such qualitative CT features to predict STAS.

Radiomics refers to extracting a large number of quantitative features from all kinds of images through predefined statistical formulas and then forming a specific model through the steps of data preprocessing, feature screening, statistical calculation, and so on (16). Studies have shown that radiology has great potential in distinguishing invasive lung cancer, and predicting treatment response (17, 18), recurrence, and metastasis (19, 20), but there are few studies on the preoperative prediction of STAS in lung adenocarcinoma. Chen et al. (9) suggested that radiomic features of the tumor body could effectively predict STAS of stage I lung adenocarcinoma preoperatively, thus assisting in the formulation of the best surgical plan. Jiang et al. (21) found that the AUC value of the radiomics model in predicting STAS was 0.754 (sensitivity, 0.880; specificity 0.588). However, these studies were based on the extraction and analysis of intranodular radiomic features and were not combined with those of perinodular features.

Although most studies have defined STAS as micropapillary clusters, solid nests, or individual cells around the main body of the tumor, its distance from the main body of the tumor has not been clearly defined (22). Various studies use the distance from the first alveoli outside the edge of the tumor, or several alveolar cavities from the main body of the tumor, or at least 0.5 mm from the edge of the main body of the tumor to define STAS. Masaik et al. (23) found that STAS positivity and a cutting-edge distance smaller than 10 mm were important risk factors for recurrence of early lung adenocarcinoma after wedge resection. Dai et al. (24) found that the maximum distance between the tumor island and the edge of the tumor in STAS+ lung adenocarcinoma was 1.35 cm. However, in a previous morphological study of STAS+ adenocarcinoma, we found that tumor cell clusters in stage I lung adenocarcinoma could spread to the alveolar cavity of the adjacent lobe through the congenital defect of the interlobar fissure.

Therefore, in this study, radiomic features at different perinodular distances within 20 mm were extracted and combined with modeling analysis. It was found that the combined intra- and perinodular model was more effective in the predictive performance of STAS+ lung adenocarcinoma than the intranodular model alone; the AUC and accuracy in the verification set were increased by 7.4 and 2.8%, respectively, and the prediction efficiencies of the two radiomics models were higher than those of traditional morphological features. Zhuo et al. (25) predicted STAS by combining morphological and radiomic features around the tumor to predict STAS. The radiomic model was established by using point localization and region growth methods to extract 5, 10, and 15 mm distances from the tumor surface, but for tumors close to the chest wall and mediastinum, this method could not avoid areas outside the lung tissue. For irregular lung adenocarcinoma, simple spherical dilatation would cause an uneven extraction area around the tumor. In this study, we wrote a program to capture the perinodular area and exclude extrapulmonary tissues; the radiomics model combining intra- and perinodular areas predicted an AUC of 0.835 and an accuracy of 0.818, which was lower than that of Zhuo et al. (25) (AUC = 0.99).

Our study proved that the combined radiomics model can significantly enhance the predictive performance of STAS. In this study, we explored the performance of different types of radiomic features and found that the radiomic model of grayscale texture features performed worst, and the intra- and perinodular model combining three types of features had the best predictive performance, which showed that imaging features can effectively predict the expression of STAS in lung cancer.

Our study had some limitations. First, manipulation or stapling devices during operation may artificially cause STAS, which is impossible to avoid in our study. Second, considering that pathologists are not trained to look for these cells in the lung parenchyma beyond the edge of the tumor, STAS is mostly overlooked on microscopic review. Finally, the combination of clinical semantics may further improve the performance of our model.

In conclusion, we introduced a machine learning approach that demonstrates the utility of combining texture features of intranodules and their surrounding lung parenchyma on non-contrast chest CT images to discriminate STAS+ and STAS− lung adenocarcinoma. Incorporation of different perinodular texture features with intranodular texture improved the predictive ability of the radiomics model to distinguish STAS+ adenocarcinoma before surgery.

## Data Availability Statement

The raw data supporting the conclusions of this article will be made available by the authors without undue reservation.

## Ethics Statement

Written informed consent was obtained from the individual(s) for the publication of any potentially identifiable images or data included in this article.

## Author Contributions

LQ: writing and study design. LH: data analysis. GC: data analysis. YC: pathological analysis. KX: writing and study design. ML: manuscript revision and data collection. XL: provided external verification data. All authors contributed to the article and approved the submitted version.

## Funding

This study was supported by the National Natural Science Foundation of China (61976238); National Natural Science Foundation of China (82071897); Research Fund of Huadong Hospital (2019lc008); Shanghai Municipal Health Commission (20204Y0299); Medical Imaging Key Program of Wise Information Technology of 120, Health Commission of Shanghai 2018ZHYL0103 (ML), “Future Star” of famous doctors’ training plan of Fudan University.

## Conflict of Interest

Authors LH and GC was employed by company Jianpei Technology Co., Ltd.

The remaining authors declare that the research was conducted in the absence of any commercial or financial relationships that could be construed as a potential conflict of interest.
