# Supplementary material for: Comparison of Diagnostic Performance of Spread Through Airspaces of Lung Adenocarcinoma Based on Morphological Analysis and Perinodular and Intranodular Radiomic Features on Chest CT Images
Source: Front Oncol. 2021 Jun 25;11:654413. doi: 10.3389/fonc.2021.654413 (PMC8268002; doi:10.3389/fonc.2021.654413)
Supplement: Supplementary file 1 [file Table_1.docx]

Table E1 The rank of selected features in 8-VOI Model and VOI_core_ Model

| model | feature class | mode | AUC | AUC 95% CIs | Acc | Sen | Spe | PPV | NPV |
| --- | --- | --- | --- | --- | --- | --- | --- | --- | --- |
| VOI core Model | First Order and Shape Features(n=18) | train | 0.852 | [0.781-0.917] | 0.820 | 0.761 | 0.841 | 0.636 | 0.906 |
|  |  | test | 0.818 | [0.636-0.964] | 0.796 | 0.700 | 0.824 | 0.539 | 0.903 |
|  | Gray Level Features(n=89) | train | 0.715 | [0.642-0.783] | 0.663 | 0.826 | 0.603 | 0.432 | 0.905 |
|  |  | test | 0.788 | [0.630-0.903] | 0.727 | 0.900 | 0.677 | 0.450 | 0.958 |
|  | Wavelet Features(n=744) | train | 0.825 | [0.745-0.895] | 0.831 | 0.739 | 0.865 | 0.667 | 0.901 |
|  |  | test | 0.829 | [0.682-0.953] | 0.818 | 0.700 | 0.853 | 0.583 | 0.906 |
|  | The Above Features(n=851) | train | 0.843 | [0.772-0.908] | 0.831 | 0.739 | 0.865 | 0.667 | 0.901 |
|  |  | test | 0.835 | [0.682-0.963] | 0.818 | 0.700 | 0.853 | 0.583 | 0.906 |
| 8-VOI Model | First Order and Shape Features(n=144) | train | 0.853 | [0.785-0.918] | 0.843 | 0.696 | 0.897 | 0.711 | 0.890 |
|  |  | test | 0.827 | [0.675-0.957] | 0.818 | 0.600 | 0.882 | 0.600 | 0.882 |
|  | Gray Level Features(n=712) | train | 0.845 | [0.779-0.907] | 0.738 | 0.913 | 0.675 | 0.506 | 0.955 |
|  |  | test | 0.782 | [0.584-0.953] | 0.818 | 0.600 | 0.882 | 0.600 | 0.882 |
|  | Wavelet Features(n=5952) | train | 0.905 | [0.859-0.951] | 0.802 | 0.913 | 0.762 | 0.583 | 0.960 |
|  |  | test | 0.877 | [0.725-0.985] | 0.841 | 0.800 | 0.853 | 0.615 | 0.936 |
|  | The Above Features(n=6808) | train | 0.907 | [0.862-0.948] | 0.831 | 0.848 | 0.825 | 0.639 | 0.937 |
|  |  | test | 0.897 | [0.784-0.985] | 0.841 | 0.900 | 0.824 | 0.600 | 0.966 |
