# Supplementary material for: Comparison of Diagnostic Performance of Spread Through Airspaces of Lung Adenocarcinoma Based on Morphological Analysis and Perinodular and Intranodular Radiomic Features on Chest CT Images
Source: Front Oncol. 2021 Jun 25;11:654413. doi: 10.3389/fonc.2021.654413 (PMC8268002; doi:10.3389/fonc.2021.654413)
Supplement: Supplementary file 2 [file Table_2.docx]

Table E2 The rank of selected features in 8-VOI Model and VOI _core_ Model

| **Rank** | **8-VOI Model** | **VOI _core_ Model** |
| --- | --- | --- |
| 1 | original_firstorder_90Percentile_org | original_firstorder_90Percentile_org |
| 2 | original_firstorder_Skewness_org | original_firstorder_Maximum_org |
| 3 | wavelet-HHH_glszm_SmallAreaLowGrayLevelEmphasis_org | original_firstorder_Mean_org |
| 4 | wavelet-HHL_firstorder_Mean_org | original_firstorder_Skewness_org |
| 5 | wavelet-HHL_firstorder_Median_org | wavelet-HLL_gldm_LargeDependenceHighGrayLevelEmphasis_org |
| 6 | wavelet-LLH_firstorder_Skewness_2mm | wavelet-LLL_firstorder_Maximum_org |
| 7 | wavelet-LLL_firstorder_Skewness_2mm | wavelet-LLL_glcm_ClusterShade_org |
| 8 | original_firstorder_Skewness_4mm | wavelet-LLL_gldm_DependenceVariance_org |
| 9 | wavelet-HLL_firstorder_Median_4mm | wavelet-LLL_gldm_LargeDependenceHighGrayLevelEmphasis_org |
| 10 | original_firstorder_90Percentile_6mm | wavelet-LLL_gldm_LowGrayLevelEmphasis_org |
| 11 | wavelet-HHH_glcm_Imc1_6mm | wavelet-LLL_glrlm_LongRunHighGrayLevelEmphasis_org |
| 12 | wavelet-LHH_firstorder_Mean_6mm | wavelet-LLL_glrlm_LongRunLowGrayLevelEmphasis_org |
| 13 | wavelet-HHH_firstorder_Mean_8mm |  |
| 14 | wavelet-HHH_glcm_Imc1_8mm |  |
| 15 | wavelet-HHH_gldm_LargeDependenceHighGrayLevelEmphasis_8mm |  |
| 16 | wavelet-HHH_firstorder_Mean_10mm |  |
| 17 | wavelet-HHH_glcm_Imc1_10mm |  |
| 18 | wavelet-LHL_firstorder_Mean_10mm |  |
| 19 | wavelet-HHH_glcm_Imc1_20mm |  |
| 20 | wavelet-HLL_firstorder_Skewness_20mm |  |
